# A Signature of Autophagy-Related Long Non-coding RNA to Predict the Prognosis of Breast Cancer

**DOI:** 10.3389/fgene.2021.569318

**Published:** 2021-03-16

**Authors:** Xiaoping Li, Jishang Chen, Qihe Yu, Hui Huang, Zhuangsheng Liu, Chengxing Wang, Yaoming He, Xin Zhang, Weiwen Li, Chao Li, Jinglin Zhao, Wansheng Long

**Affiliations:** ^1^Department of Gastrointestinal Surgery, Affiliated Jiangmen Hospital of Sun Yat-sen University, Jiangmen, China; ^2^Department of Breast Surgery, Yangjiang People's Hospital, Yangjiang, China; ^3^Department of Oncology, Affiliated Jiangmen Hospital of Sun Yat-sen University, Jiangmen, China; ^4^Department of Breast Surgery, Jiangmen Maternity & Chile Health Care Hospital, Jiangmen, China; ^5^Department of Radiology, Affiliated Jiangmen Hospital of Sun Yat-sen University, Jiangmen, China; ^6^Clinical Experimental Center, Jiangmen Key Laboratory of Clinical Biobanks and Translational Research, Affiliated Jiangmen Hospital of Sun Yat-sen University, Jiangmen, China; ^7^Department of Breast and Thyroid Surgery, Affiliated Jiangmen Hospital of Sun Yat-sen University, Jiangmen, China

**Keywords:** breast cancer, autophagy, long non-coding RNA, nomogram, gene set enrichment analysis

## Abstract

**Background:** A surge in newly diagnosed breast cancer has overwhelmed the public health system worldwide. Joint effort had beed made to discover the genetic mechanism of these disease globally. Accumulated research has revealed autophagy may act as a vital part in the pathogenesis of breast cancer.

**Objective:** Aim to construct a prognostic model based on autophagy-related lncRNAs and investigate their potential mechanisms in breast cancer.

**Methods:** The transcriptome data and clinical information of patients with breast cancer were obtained from The Cancer Genome Atlas (TCGA) database. Autophagy-related genes were obtained from the Human Autophagy Database (HADb). Long non-coding RNAs (lncRNAs) related to autophagy were acquired through the Pearson correlation analysis. Univariate Cox regression analysis as well as the least absolute shrinkage and selection operator (LASSO) regression analysis were used to identify autophagy-related lncRNAs with prognostic value. We constructed a risk scoring model to assess the prognostic significance of the autophagy-related lncRNAs signatures. The nomogram was then established based on the risk score and clinical indicators. Through the calibration curve, the concordance index (C-index) and receiver operating characteristic (ROC) curve analysis were evaluated to obtain the model's predictive performance. Subgroup analysis was performed to evaluate the differential ability of the model. Subsequently, gene set enrichment analysis was conducted to investigate the potential functions of these lncRNAs.

**Results:** We attained 1,164 breast cancer samples from the TCGA database and 231 autophagy-related genes from the HAD database. Through correlation analysis, 179 autophagy-related lncRNAs were finally identified. Univariate Cox regression analysis and LASSO regression analysis further screened 18 prognosis-associated lncRNAs. The risk scoring model was constructed to divide patients into high-risk and low-risk groups. It was found that the low-risk group had better overall survival (OS) than those of the high-risk group. Then, the nomogram model including age, tumor stage, TNM stage and risk score was established. The evaluation index (C-index: 0.78, 3-year OS AUC: 0.813 and 5-year OS AUC: 0.785) showed that the nomogram had excellent predictive power. Subgroup analysis showed there were difference in OS between high-risk and low-risk patients in different subgroups (stage I-II, ER positive, Her-2 negative and non-TNBC subgroups; all *P* < 0.05). According to the results of gene set enrichment analysis, these lncRNAs were involved in the regulation of multicellular organismal macromolecule metabolic process in multicellular organisms, nucleotide excision repair, oxidative phosphorylation, and TGF-β signaling pathway.

**Conclusions:** We identified 18 autophagy-related lncRNAs with prognostic value in breast cancer, which may regulate tumor growth and progression in multiple ways.

## Introduction

Breast cancer is the most common malignant tumor and the second leading cause of cancer death in females. In 2018, there were ~2.08 million new cases and 630,000 deaths worldwide (Bray et al., 2018). Accurately predicting the prognosis of breast cancer is essential for improving the prognosis and providing appropriate treatment for patients. With the advancement of high-throughput technologies, various prognostic models have been developed according to the sequencing data. Moreover, prognostic models based on the autophagy-related genes, immune-related genes, or immune-related lncRNAs have been studied before (Li B. et al., 2020; Lin et al., 2020; Shen et al., 2020). But none of them combined clinical data with sequencing data to build a predictive model for breast cancer patients. Our research aims to establish a prognostic model for patients with breast cancer utilizing the autophagy-related lncRNAs and clinical data, which may serve as an important supplement to prognosis prediction.

Long non-coding RNA (lncRNA) is a type of RNA with more than 200 nucleotides, which functions by regulating gene expression and interacting with specific proteins (Esteller, 2011; Kopp and Mendell, 2018; Choudhari et al., 2020). Substantial evidence showed that lncRNAs were involved in a series of biological processes, including proliferation and differentiation of tumor cells, chromosome remodeling, transcription, as well as post-transcriptional modification (Wang and Chang, 2011; Lau, 2014; Tomar et al., 2020; Wu et al., 2020). The role of lncRNA in breast cancer has been revealed gradually (Wang et al., 2017; Richard and Eichhorn, 2018). Wang et al. (2020) found that lncRNA-encoded polypeptide ASRPS could inhibit breast cancer angiogenesis. Dong et al. (2019) demonstrated that lncRNA TINCR promoted chemotherapy resistance and epithelial-mesenchymal transition of breast cancer by targeting microRNA-125b. Previous studies suggested that lncRNA has a potential impact on the prognosis and targeted therapy of breast cancer (Jiang et al., 2016; Yang et al., 2018; Du et al., 2019).

Autophagy is a cellular process in which damaged organelles and unnecessary macromolecules were degraded and recycled under the control of autophagy-related genes (Li X. et al., 2020), which played a unique role in different stages of tumor pathogenesis. Previous studies disclosed that decreased expression of autophagy-related gene Beclin-1 could inhibit autophagy of tumor cells and further accelerate breast cancer progression (Lisiak et al., 2018; Hamurcu et al., 2019). Wei et al. (2020) indicated that Magnoflorine could induce autophagy in breast cancer cells through the AKT/mTOR signaling pathway, and thus increasing their sensitivity to doxorubicin. Lin et al. (2020) proved that the prognostic model of breast cancer based on autophagy-related genes has satisfactory predictive power. Taken together, cell autophagy plays an important role in the progression, treatment, and prognosis of breast cancer.

Autophagy-related lncRNAs may have a potential effect on the prognosis of breast cancer patients. It has been reported that lncRNA H19 induces the tamoxifen-resistance in breast cancer through the activation of the H19/SAHH/DNMT3B axis, thereby facilitating cell autophagy (Wang et al., 2019). Both *in vitro* and *in vivo* experiments indicated that lncRNA NAMPT-AS regulated tumor cell autophagy and promoted cancer progression through the mTOR signaling pathway (Zhang et al., 2019). Luan et al. (2019) screened 10 autophagy-related lncRNAs in glioma and proved the prognostic value of these lncRNAs. This paper aims to construct a prognostic model based on autophagy-related lncRNAs and investigate their potential mechanisms in breast cancer.

## Materials and Methods

### Sample Resources and Processing

**Figure 1:**
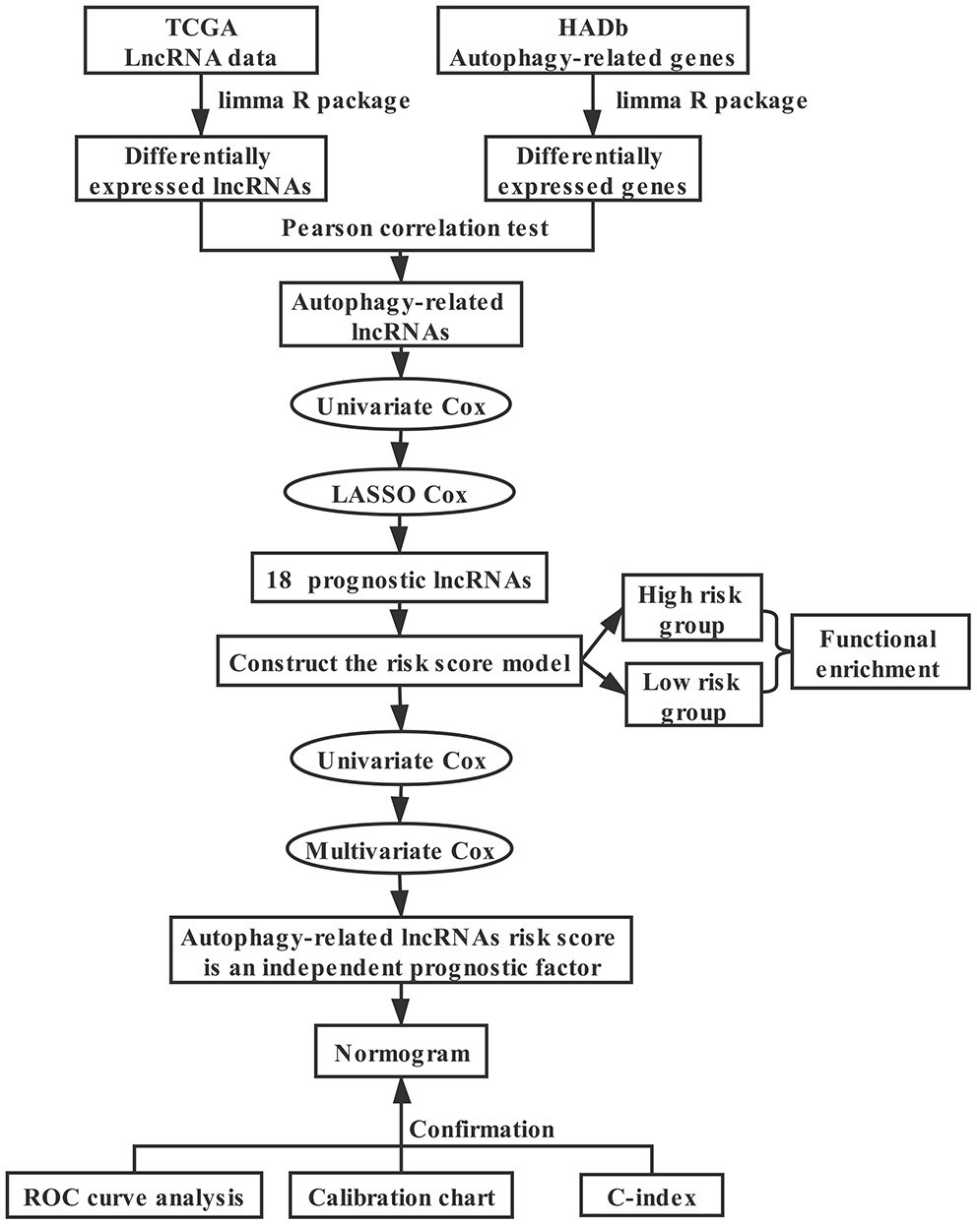
Flow chart. Flowchart for establishing and evaluating the prognostic model.

The technical roadmap for the development of an autophagy-related lncRNA breast cancer prognosis model is shown in Figure 1. We downloaded the transcriptome data including mRNA and lncRNA as well as corresponding clinical information of breast cancer from the Cancer Genome Atlas database (TCGA, https://cancergenome.nih.gov/), and autophagy-related genes from the human autophagy database (HADb, http://www.autophagy.lu/). We used | log2FC | > 0.5 and false discovery rate (FDR) < 0.05 as the thresholds to identify differentially expressed autophagy-related genes and lncRNAs between cancer samples and normal samples. Pearson correlation analysis was utilized to calculate the correlation coefficient (*R*^2^) between lncRNAs and autophagy-related genes. Also, lncRNAs with | *R*^2^ | > 0.4 and *p* < 0.05 were defined as autophagy-related lncRNAs.

### Construction of Risk Scoring Model

Univariate Cox regression analysis was performed on autophagy-related lncRNAs, and prognosis-related lncRNAs were recognized with criteria of *p* < 0.05. We performed the LASSO regression analysis to identify the crucial lncRNAs that were closely associated with OS. The selected lncRNAs were included in the multivariate Cox regression model to generate their coefficients (coefi). A risk scoring model containing both coefi and lncRNA expression levels (expi) was constructed to get the risk score for all samples. Patients were divided into high-risk group and low-risk group with the median risk score as the cut-off point. What's more, survival curve was executed to compare the prognosis of the two groups. Univariate and multivariate Cox regression analysis were exploited to evaluate the prognostic significance of risk score and clinical factors including age, tumor stage, and TNM stage. The area under the curve (AUC) was generated by ROC curve analysis to assess the predictive effectiveness of the model. Then we further evaluated the model by stratifying the patients into different subgroups by clinical stage, ER status, HER-2 status and TNBC.

### Establishment and Evaluation of Prognostic Model

We established a nomogram based on the multivariate Cox regression results of age, tumor stage, TMN stage, and risk score. The 3- and 5-year OS of each patient was predicted based on the nomogram. Meanwhile, the concordance index (C-index), calibration curve, and ROC curve were generated to evaluate the predicted efficacy of the model.

### Gene Set Enrichment Analysis

Gene Ontology (GO) enrichment analysis and Kyoto Encyclopedia of Genes and Genomes (KEGG) pathway analysis were applied to differentially expressed genes between high-risk and low-risk groups. We also analyzed the gene set to deduce their functions and determined whether the gene set differed significantly between the two groups. The research was to investigate whether the differentially expressed genes between the two groups were enriched during autophagy.

## Results

### Differentially Expressed Autophagy-Related Genes and lncRNAs

**Figure 2:**
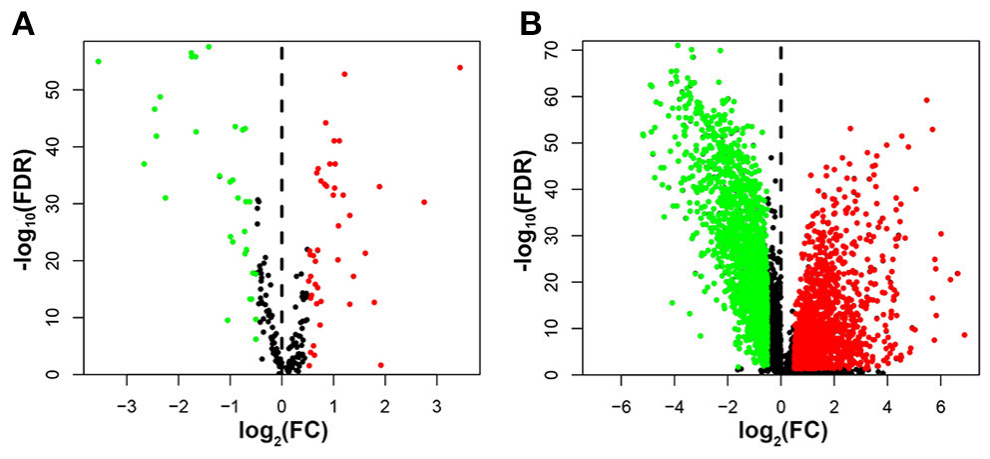
The volcano plot showed differential expression of autophagy-related genes and lncRNAs in breast cancer tissues and adjacent tissues. Red represented significantly up-regulated lncRNAs and autophagy genes, green showed significantly down-regulated ones, and black indicated no different ones. **(A)** The volcano plot demonstrating the differentially expressed autophagy-related genes. **(B)** The volcano plot demonstrating the differentially expressed lncRNAs.

We extracted breast cancer samples from the TCGA database, including 1,053 tumor tissues and 111 normal tissues, and 231 autophagy-related genes were generated from the HADb database. Differentially expressed autophagy-related genes and lncRNAs were identified between breast cancer and para-cancer tissues with the help of the “limma” package in the R software. Seventy-five differentially expressed autophagy-related genes were found, of which 32 genes were down-regulated and 43 genes were up-regulated (Figure 2A). Besides, we recognized 3,770 differentially expressed lncRNAs with 2,068 lncRNAs down-regulated and 1,702 lncRNAs up-regulated (Figure 2B). Then, we attained 179 autophagy-related lncRNAs through Pearson correlation analysis with criteria of | *R*^2^ | > 0.4 and *p* < 0.05 (Supplementary Table 1).

### Construction of Risk Scoring Model

**Figure 3:**
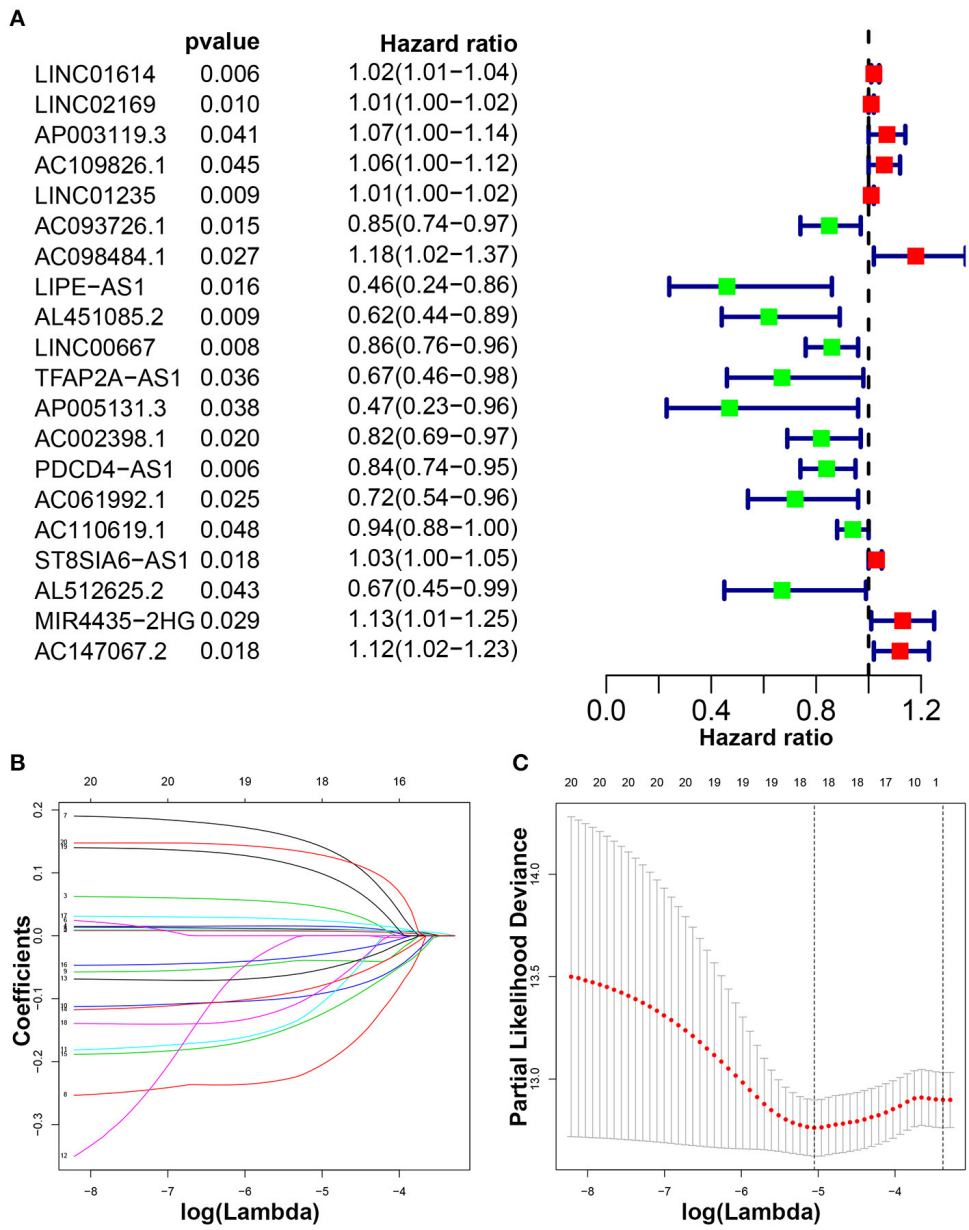
Identification of autophagy-related lncRNAs with prognostic value in breast cancer. **(A)** The risk ratio forest plot showed that 20 autophagy-related lncRNAs were significantly related to the OS. **(B)** Adjusted parameters of LASSO regression model. **(C)** Illustration for LASSO coefficient spectrum of prognostic lncRNAs.

**Figure 4:**
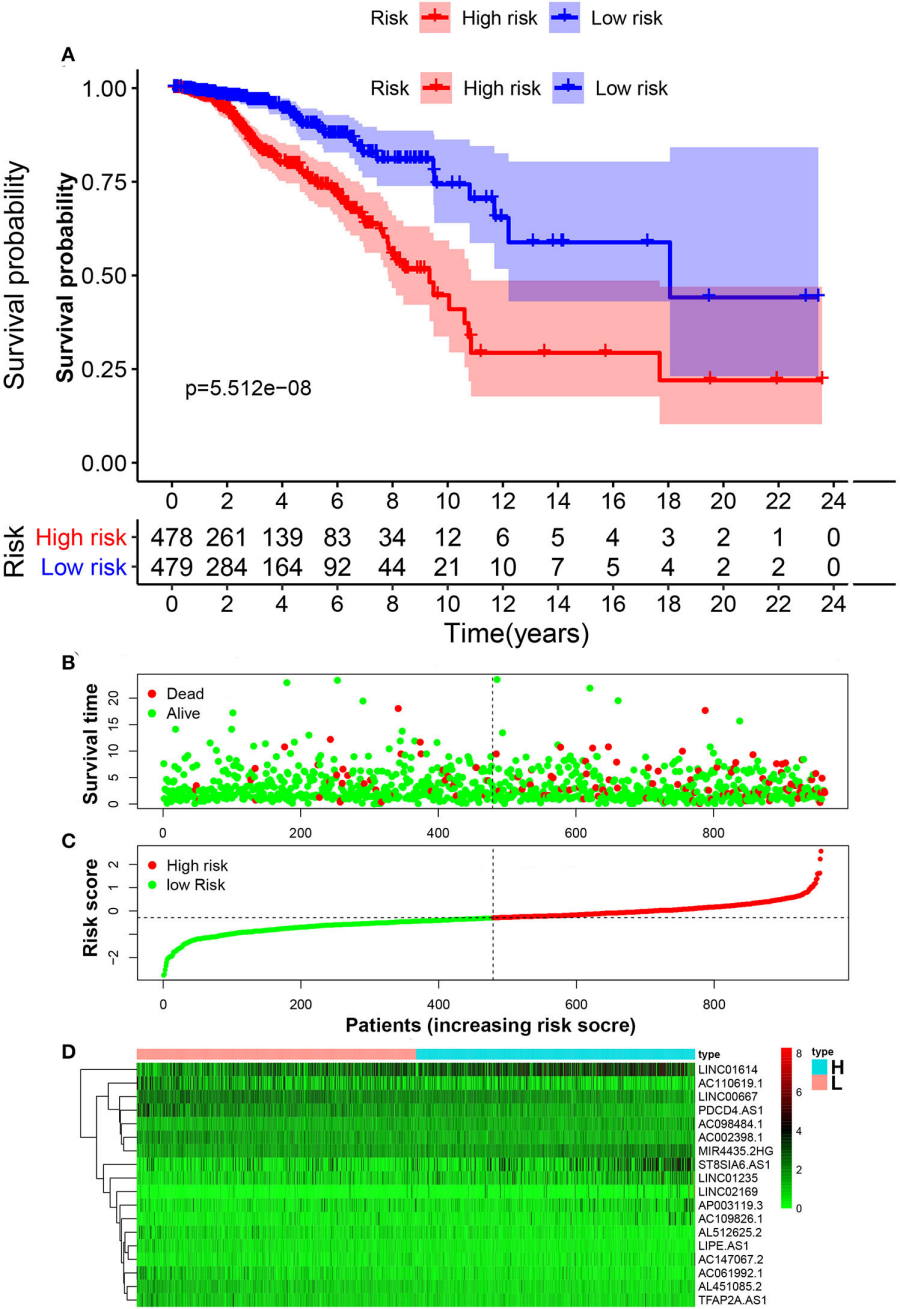
Construction of risk scoring model. **(A)** Kaplan–Meier survival analysis of breast cancer patients showed that the high-risk group had significantly worse OS than that in low-risk group. **(B)** The survival rate and survival status of breast cancer patients. **(C)** The distribution of 18-lncRNA risk scores for each patient. **(D)** Heatmap of 18 lncRNAs in both low-risk group and high-risk group.

**Figure 5:**
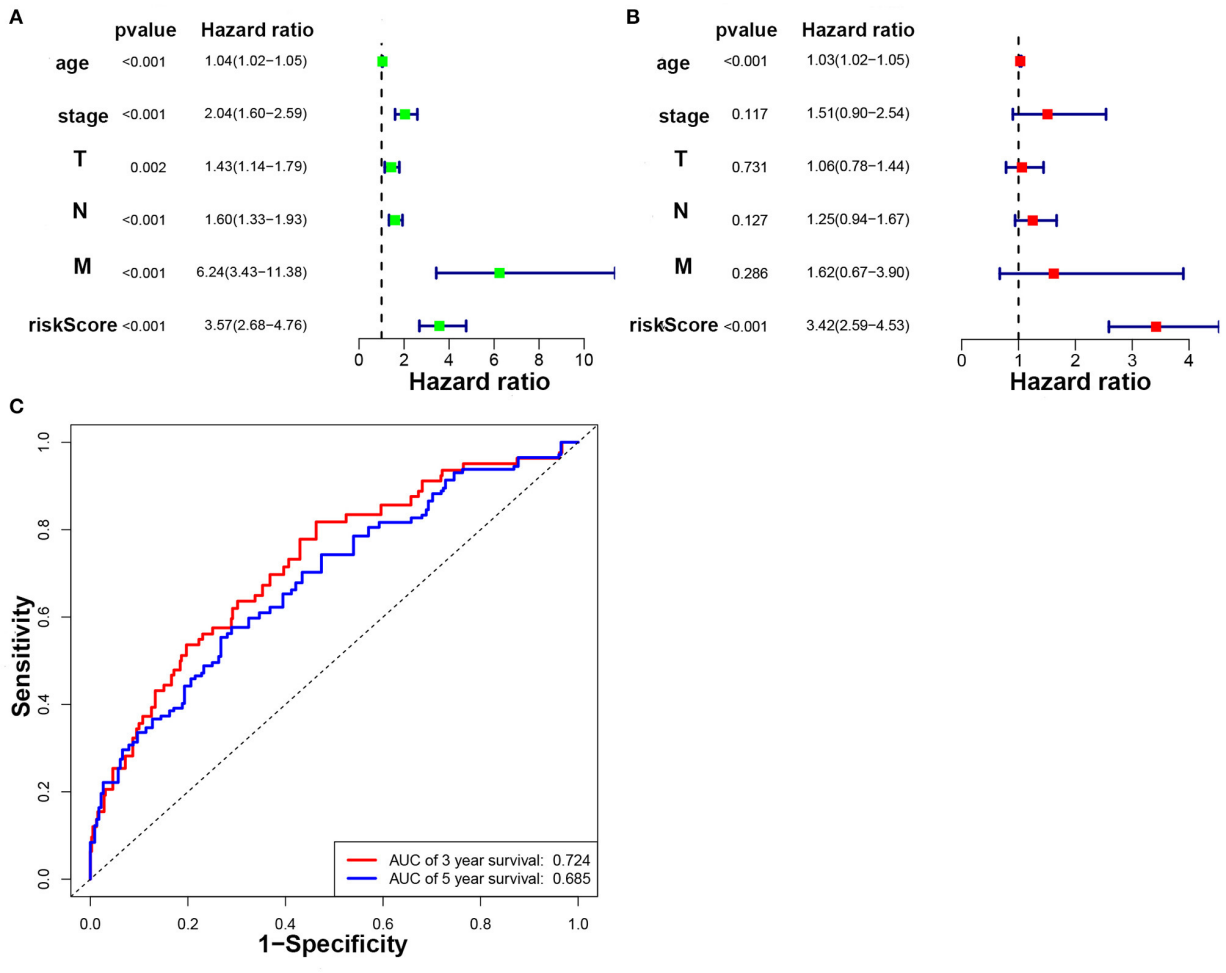
The prognostic value of clinical pathological characteristics and risk scores. **(A)** Univariate Cox regression analysis of breast cancer patients. **(B)** Multivariate Cox regression analysis of breast cancer patients. **(C)** ROC curve analysis of breast cancer prognosis model based on autophagy-related lncRNAs.

**Figure 6:**
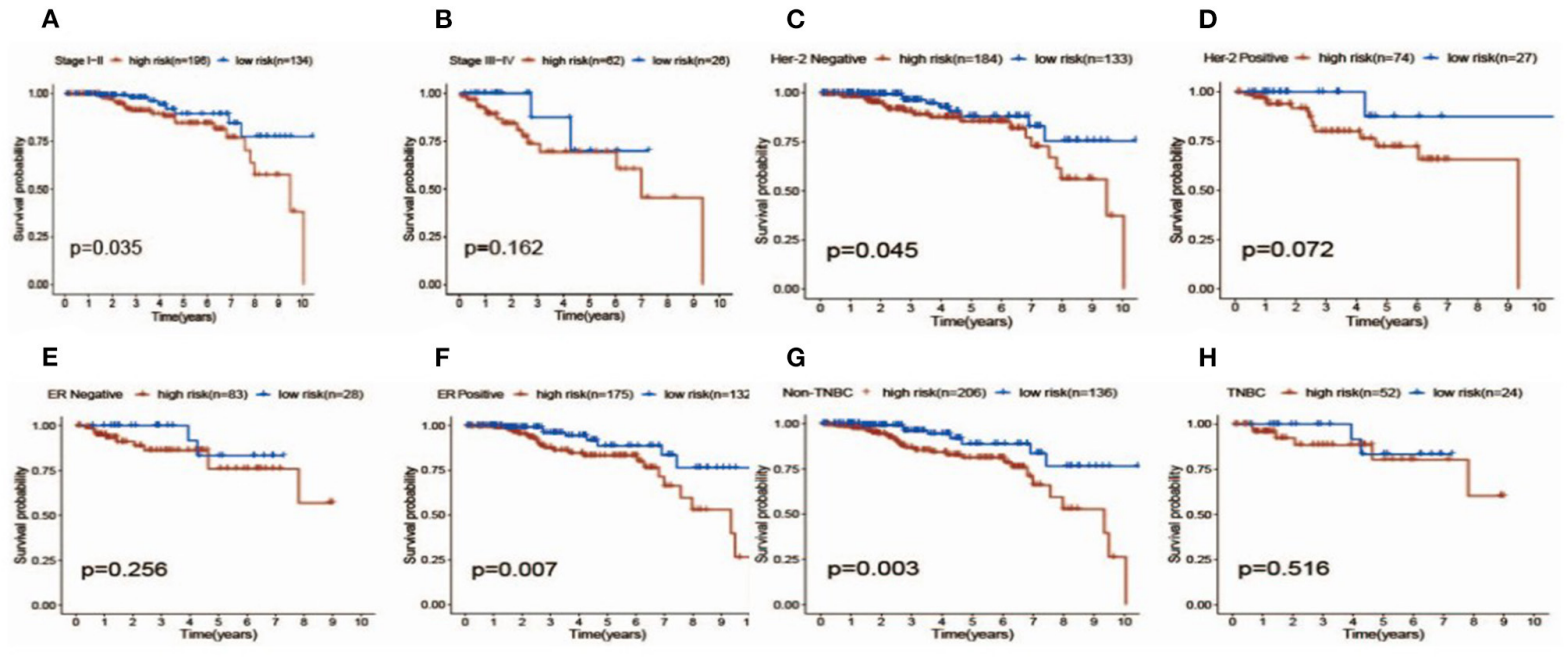
Survival analysis of patients with high (red) vs. Low (blue) risk scores in different subgroups including different stages **(A,B)**, her-2 status **(C,D)**, ER status **(E,F)** and TNBC status **(G,H)**.

Patients with follow-up time exceeding 30 days were included in univariate Cox regression analysis to explore the prognostic significance of autophagy-related lncRNAs. Twenty prognosis-associated lncRNAs were acquired with *p* < 0.05 (Figure 3A). We further screened 18 core lncRNAs tightly related to prognosis by LASSO regression analysis (Figures 3B,C). The multivariate Cox regression was employed to construct the risk scoring model as follows: Risk score $=\overset{18}{\underset{i=1}{\sum }}\left(coefi*expi\right)$. Patients were divided into high-risk group and low-risk group with the median risk score as the cut-off point. Survival curve result showed that the OS in the low-risk group was significantly higher than that in the high-risk group (*p* < 0.0001, Figure 4A). The risk curve and scatterplot showed that the low-risk group had lower risk coefficient and mortality rate compared to the high-risk group (Figures 4B,C). The heat map visualized the expression levels of the 18-lncRNAs between the high-risk and low-risk groups (Figure 4D). Univariate and multivariate Cox regression analysis results showed that 18-lncRNAs signature was an independent prognostic factor of patients with breast cancer (Figures 5A,B). The AUCs of 3- and 5-year OS generated by ROC curve analysis were 0.724 and 0.685, respectively (Figure 5C). In subgroup analysis, the risk scoring model showed satisfying differential ability in stage I-II, ER positive, Her-2 negative, and non-TNBC subgroups (*P* < 0.05). Due to small sample size of stage III-IV, ER negative, Her-2 positive and TNBC subgroups, the survival curve showed potential identification trends, but there were no statistical difference (Figure 6).

### Establishment and Evaluation of the Prognostic Model

**Figure 7:**
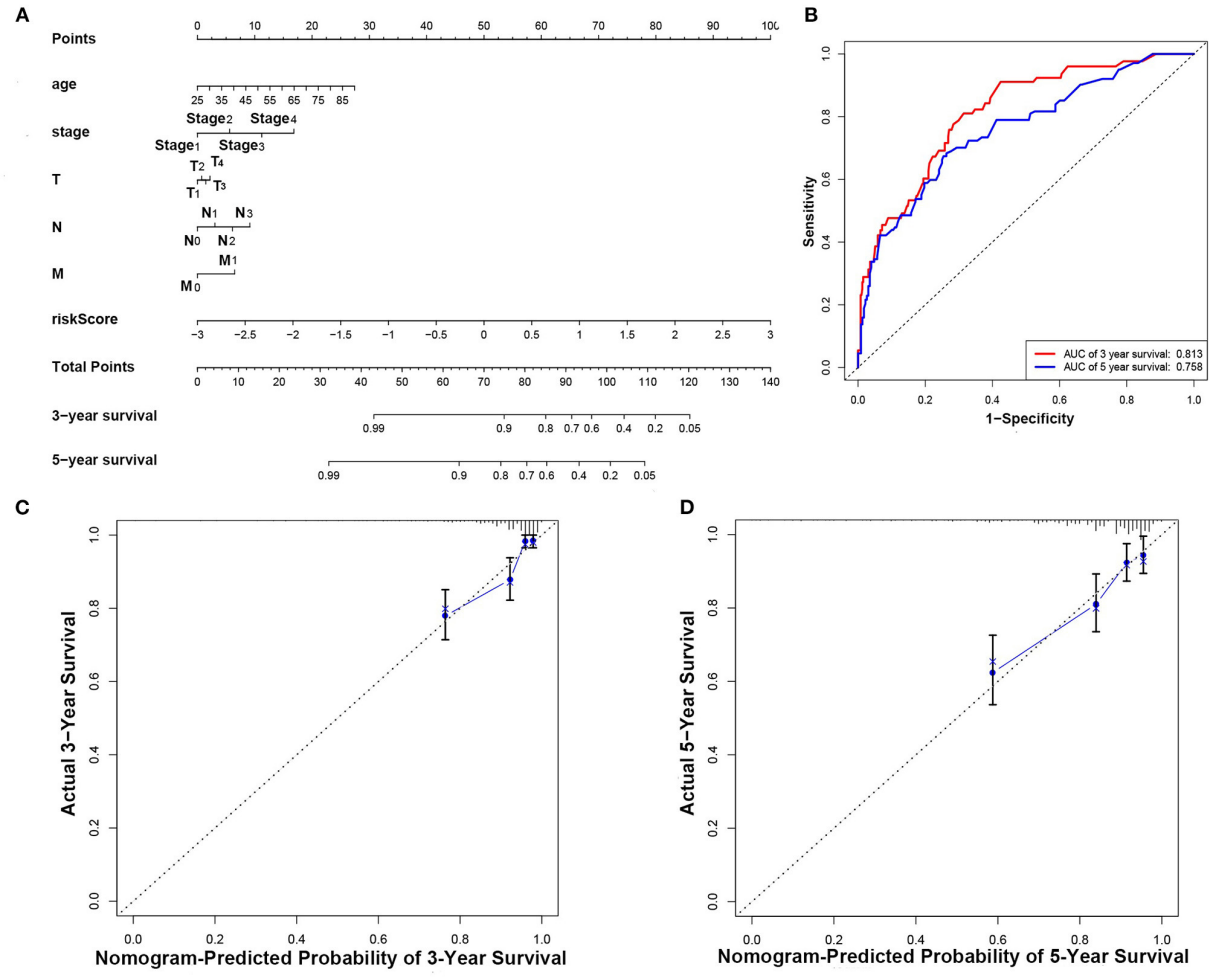
Construction and evaluation of the nomogram. **(A)** Nomogram for predicting the 3- and 5-year survival rates of patients with breast cancer. **(B)** ROC curve analysis demonstrated that AUCs for 3- and 5-year OS predicted by the nomogram were 0.813 and 0.785, respectively. **(C)** Calibration chart based on 3-year OS of the nomogram. **(D)** Calibration chart based on 5-year OS of the nomogram.

The nomogram was established according to the results of multivariate Cox regression including age, tumor stage, TMN stage, and risk score (Figure 7A). The calibration curve was shown in Figure 7. The C-index of the nomogram was 0.78. And the AUCs of the 3- and 5-year OS were 0.813 and 0.785, respectively (Figure 7B). Our results showed that the prognostic model presented excellent accuracy on prediction (Figures 7C,D).

### Gene Set Enrichment Analysis

**Figure 8:**
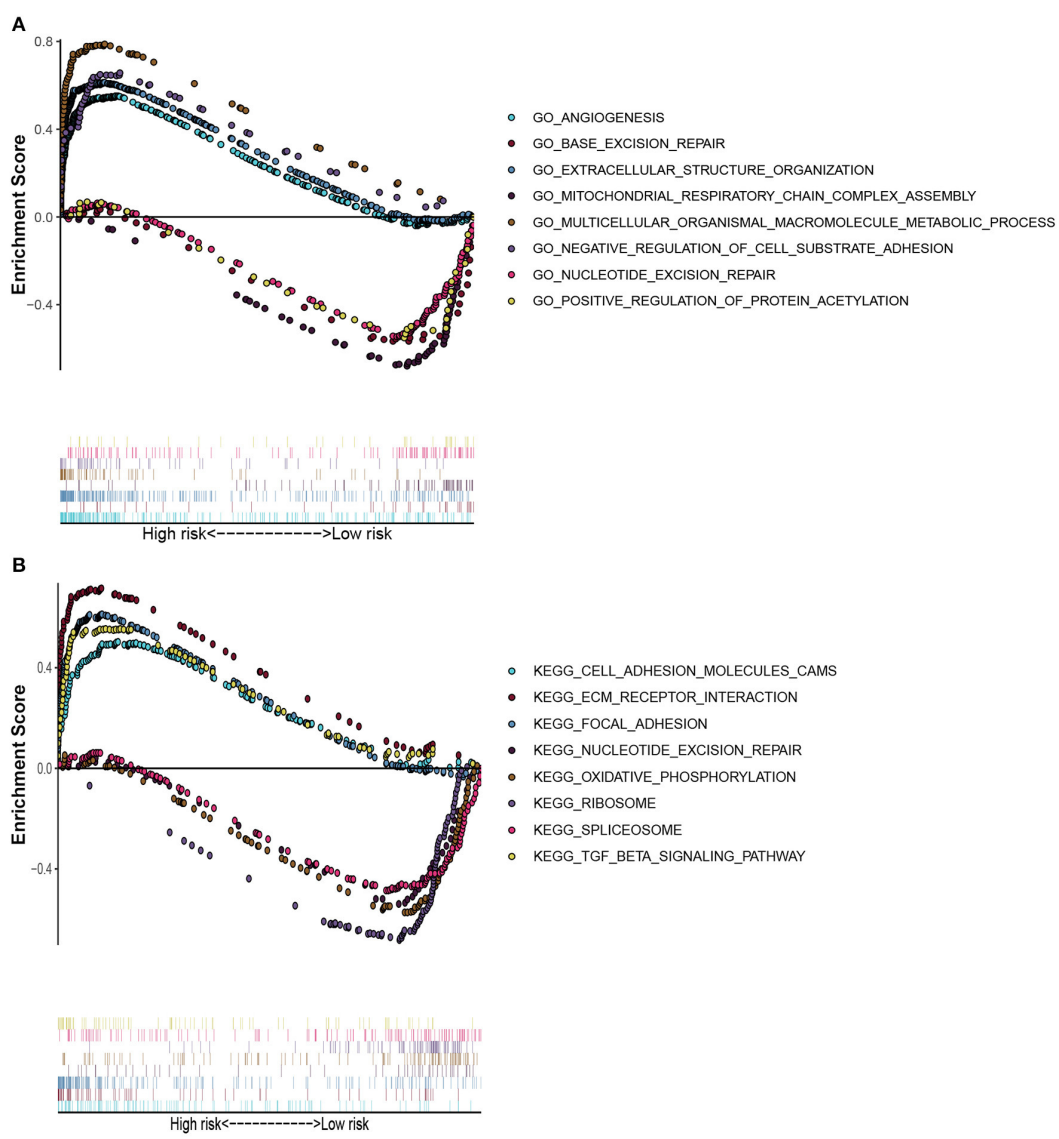
Gene set enrichment analysis. **(A)** GO enrichment analysis showed that these genes were enriched in angiogenesis, base excision repair, extracellular structural organization, mitochondrial respiratory chain complex assembly, multicellular organismal macromolecule metabolism, negative regulation of cell-substrate adhesion, nucleotide excision repairment, and positive regulation of protein acetylation. **(B)** KEGG pathway analysis indicated that these genes were involved in cams of cell adhesion molecules, ECM receptor interaction, focal adhesion, nucleotide excision repairment, oxidative phosphorylation, ribosome, spliceosome, and TGF-β signaling pathway. These contents may assist researchers to further explore the mechanisms of autophagy-related lncRNAs influencing breast cancer pathogenesis.

GO enrichment analysis and KEGG pathway analysis were conducted on differentially expressed genes between high-risk and low-risk groups. GO enrichment analysis indicated that the genes were enriched in angiogenesis, base excision repair, extracellular structure organization, assembly of mitochondrial respiratory chain complex, multicellular biological macromolecule metabolic process, negative regulation of cell-substrate adhesion, nucleotide excision repair, and positive regulation of protein acetylation (Figure 8A). KEGG pathway analysis denoted that these genes were involved in the modulation of cell adhesion molecules cams, ECM receptor interaction, focal adhesion, nucleotide excision repair, oxidative phosphorylation, ribosome, spliceosome, and TGF-β signaling pathway. All these contents may assist researchers to further explore the mechanisms of autophagy-related lncRNAs influencing breast cancer pathogenesis (Figure 8B).

## Discussion

The incidence of newly diagnosed breast cancer is increasing, which severely affect women's life. Zhou et al. (2017) demonstrated that genetic variation of autophagy-related genes was associated with the prognosis of breast cancer patients. Autophagy can maintain the homeostasis and the survival of breast cancer cells by removing dysfunctional or unnecessary substances (Han et al., 2018; Ostendorf and Tavazoie, 2020). In *vitro* experiments showed that inhibiting autophagy could hinder the survival and metastasis of dormant breast cancer cells (Vera-Ramirez et al., 2018). Meanwhile, previous researchers have suggested that autophagy was tightly related to breast cancer prognosis (Gu et al., 2016; Lin et al., 2020; Zhong et al., 2020). Hence, finding reliable autophagy-related prognostic indicators may drive for early risk stratification and proper therapeutic decision for breast cancer.

The process of autophagy can be regulated by lncRNAs in a variety of ways. Particularly, lncRNAs could adsorb autophagy-related miRNA as a molecular sponge to regulate the expression of autophagy-related genes. It was found that LncRNA H19 could induce autophagy and suppress epithelial-mesenchymal transformation in breast cancer by targeting the let-7 miRNA family (Xiong et al., 2020). LncRNA HOTAIR may affect the autophagy of breast cancer cells by sponging miR-34a (Pawlowska et al., 2017). Luen et al. showed that the risk scoring model constructed by autophagy-related lncRNAs could effectively predict the prognosis of glioma patients (Luan et al., 2019). However, the prognostic role of autophagy-related lncRNAs in breast cancer has not been studied before.

In this study, we obtained breast cancer RNA-sequence data and clinical information from the TCGA database and autophagy-related genes from the HADb database. Univariate Cox analysis and LASSO regression analysis identified 18 autophagy-related lncRNAs with prognostic significance. A risk scoring model was constructed based on these lncRNAs to divide patients into high-risk and low-risk groups. Survival analysis revealed the low-risk sub-group had much more favorable prognosis. Sequentially, we established a nomogram based on the risk scores and clinical variables including age, tumor stage, and TMN stage to predict the 3- and 5-year OS of patients. The results of the C-index, calibration curve, and ROC curve validated the predictive performance of the model. The result of subgroup analysis implicated the model may have better differential ability in hormone positive and Her-2 negative breast cancer. Further studies are required to validate the result above.

Among the 18 vital lncRNAs, 9 lncRNAs (LIPE.AS1, AC061992.1, TFAP2A.AS1, LINC00667, AL512625.2, PDCD4.AS1, AC002398.1, AL451085 2, and AC110619.1) were protective factors for the prognosis of breast cancer, while other lncRNAs (LINC02169, LINC01235, LINC01614, AC109826.1, ST8SIA6.AS1, AP003119.3, MIR4435.2HG, AC147067.2, and AC098484.1) behaved the opposite (Supplementary Table 2). Previous studies have shown that AC061992.1 and LINC00667 are protective prognostic factors for breast cancer patients (Fan et al., 2018; Zhu et al., 2020). Zhou et al. (2019) reported that lncRNA TFAP2A.AS1 could inhibit the proliferation and invasion of breast cancer cells by adsorbing miR-933. Jadaliha et al. (2018) found that lncRNA PDCD4.AS1 was capable of regulating the post-transcriptional process of oncogenes or tumor suppressor genes, thereby accelerating the progress of breast cancer. The above studies suggested that the screened lncRNAs played an important role in the progress of breast cancer.

The correlation frequency between autophagy-related genes and 18 prognosis-associated lncRNAs was shown in Supplementary Figure 1. The BAX expression in breast cancer cell line MCF-7 was upregulated when the apoptosis increasing significantly, caused by the transfection of HCCR-1 siRNA (Meng et al., 2019). Through real-time PCR, Shen et al. (2015) revealed that ATG16L2, an autophagy-related gene, was closely related to cisplatin-induced apoptosis of breast cancer cell. Numerous studies have revealed the signaling pathway of autophagy and programmed cell death (Nagakannan et al., 2016; Chang et al., 2020). However, few studies could perfectly explain the role of BAX and ATG16L2 in breast cancer treatment resistance. ITGB1 was associated with EMT of breast cancer and the ITGB1/FAK/Src/AKT/β-catenin/MMP-9 signaling axis was an important signaling pathway (Du et al., 2019). ERBB2 was a star gene. Twenty-five percent of breast cancer over-expressed ERBB2, which conferred a more aggressive phenotype and targeted therapy was needed (D'Amato et al., 2015). Carolina et al. found the chromodomain helicase CHD4 regulated ERBB2 signaling pathway and autophagy, which represented a mechanism of resistance against Trastuzumab, a therapeutic anti-ERBB2 antibody (D'Alesio et al., 2019). MLST8 that controlled the rates of cell growth and proliferation was regulated by the mTOR pathway (McMahon et al., 2005). However, the function of MLST8 and SPNS1 in breast cancer was still unknown. In brief, the intrinsic mechanism of these genes in recurrence and metastasis of breast cancer needs to be clarified in our next step of research.

To further reveal the functions of 18-lncRNAs signature in breast cancer, we executed GO enrichment analysis and KEGG pathway analysis on the differentially expressed genes between high-risk and low-risk groups. We found that these lncRNAs were involved in regulating angiogenesis, repairing nucleotide excision, oxidative phosphorylation, TGF-β signaling pathway, and assembly of the mitochondrial respiratory chain. It is worth noting that the deletion of autophagy-related gene RB1CC1 could reduce the mitochondrial mass and oxidative respiration capacity of breast tumor cells, as well as inhibit the phosphorylation of mTOR substrate (Yeo et al., 2018). Breast cancer stem cells were reported to regulate the occurrence of autophagy through the TGF-β pathway (Yeo and Guan, 2016). Autophagy inducers could suppress breast cancer angiogenesis and thus inhibit the progression of breast tumors (Li et al., 2016). In summary, our findings indicated that the 18-lncRNAs signature was essential for autophagy modulation and tumor pathogenesis. However, the specific mechanism of 18 autophagy-related lncRNAs in breast cancer still needs further elucidation in *vitro* experiments.

There are still some limitations to the study. As it is a retrospective study derived from public data, it lacks some information such as further subdivision of ER subtypes and TNBC subtypes. Though the signature provides fine predictive effect on the prognosis of hormone receptor positive subgroup, it can not further verify its predictive performance in ER alpha and ER beta subtypes. As it is a retrospective study derived from public data, it lacks some information such as recurrence time and treatment records. Also, a relatively small size cohort of TNBC patients limits the predictive performance of the model in some subgroups analysis. Further in *vivo* or in *vitro* assays and prospective clinical trials are needed to verify our conclusions.

## Conclusion

In summary, the present study constructed an 18-autophagy-related lncRNAs signature to predict the prognostic value for breast cancer patients. The prognostic model based on these lncRNAs has proved to be effective in predicting the OS. Moreover, gene set enrichment analysis revealed that the 18-lncRNAs signature might regulate the progress of breast cancer through a series of biological pathways.

## Data Availability Statement

The datasets presented in this study can be found in online repositories. The names of the repository/repositories and accession number(s) can be found in the article/Supplementary Material.

## Author Contributions

XL conceived the study, performed the data analysis, prepared the figures and tables, authored and reviewed drafts of the paper, and approved the final draft. JC collected the data from database, prepared the figures, reviewed drafts of paper and approved the final draft. JZ and WLo conceived the study, prepared the figures, reviewed drafts of the paper, and approved the final draft. All authors contributed to the article and approved the submitted version.

## Conflict of Interest

The authors declare that the research was conducted in the absence of any commercial or financial relationships that could be construed as a potential conflict of interest.
