# Supplementary material for: A Signature of Autophagy-Related Long Non-coding RNA to Predict the Prognosis of Breast Cancer
Source: Front Genet. 2021 Mar 16;12:569318. doi: 10.3389/fgene.2021.569318 (PMC8007922; doi:10.3389/fgene.2021.569318)
Supplement: Supplementary file 1 [file Table_1.DOCX]

| Table 1. Autophagy related lncRNAs. | | | |
| --- | --- | --- | --- |
| Autophagy Gene | lncRNA | cor | p-value |
| ITGB1 | LINC01614 | 0.50832144 | 2.89E-70 |
| BAX | AL451085.2 | 0.436923966 | 2.53E-50 |
| MLST8 | AL451085.2 | 0.412953348 | 1.27E-44 |
| ITGB1 | AC002398.1 | -0.409022114 | 9.87E-44 |
| BAX | AC002398.1 | 0.401755157 | 4.08E-42 |
| FADD | AP003119.3 | 0.157109732 | 2.99E-07 |
| ERBB2 | AC109826.1 | 0.223248702 | 2.33E-13 |
| CX3CL1 | AC109826.1 | 0.208446637 | 8.42E-12 |
| NRG2 | LINC01235 | 0.202781694 | 3.10E-11 |
| ITGB4 | LINC01235 | 0.315795745 | 8.16E-26 |
| PTEN | AC098484.1 | 0.296778443 | 7.45E-23 |
| ATG16L2 | LINC00667 | 0.201619721 | 4.04E-11 |
| BIRC5 | AC061992.1 | 0.336188291 | 3.09E-29 |
| FAS | AC110619.1 | -0.206719531 | 1.26E-11 |
| SPNS1 | AL512625.2 | 0.393587614 | 2.40E-40 |
| GRID1 | AC147067.2 | 0.194726917 | 1.86E-10 |
| CTSB | MIR4435.2HG | 0.193131113 | 2.63E-10 |
| SPHK1 | MIR4435.2HG | 0.245645098 | 6.17E-16 |
| VEGFA | MIR4435.2HG | 0.193443924 | 2.46E-10 |
| BAX | MIR4435.2HG | 0.272994711 | 1.88E-19 |
| CCL2 | MIR4435.2HG | 0.18605256 | 1.18E-09 |
| ERO1A | ST8SIA6.AS1 | 0.204382553 | 2.15E-11 |
| ERBB2 | ST8SIA6.AS1 | 0.241276794 | 2.06E-15 |
| ULK3 | PDCD4.AS1 | 0.183833945 | 1.86E-09 |
| CTSD | PDCD4.AS1 | 0.160831179 | 1.55E-07 |
| SPNS1 | PDCD4.AS1 | 0.151854947 | 7.40E-07 |
| BAX | PDCD4.AS1 | 0.164848293 | 7.46E-08 |
| MLST8 | PDCD4.AS1 | 0.165393195 | 6.74E-08 |
| VMP1 | TFAP2A.AS1 | 0.293876384 | 2.02E-22 |
| ATG16L2 | TFAP2A.AS1 | 0.336305457 | 2.94E-29 |
| CASP3 | LIPE.AS1 | -0.191555641 | 3.69E-10 |
| ITGB1 | LIPE.AS1 | -0.239867392 | 3.02E-15 |
| P4HB | LINC02169 | 0.144948389 | 2.32E-06 |
| CHMP4B | LINC02169 | 0.10519798 | 0.000628271 |
| CXCR4 | MIR155HG | 0.503286763 | 1.06E-68 |
| FAS | MIR155HG | 0.418958971 | 5.22E-46 |
| CASP1 | MIR155HG | 0.438592794 | 9.75E-51 |
| PRKCQ | MIR155HG | 0.671828475 | 3.64E-139 |
| CFLAR | MIR155HG | 0.435905484 | 4.52E-50 |
| ITGB1 | MIR200CHG | -0.428049827 | 3.69E-48 |
| RGS19 | MHENCR | 0.419010977 | 5.08E-46 |
| BAX | MHENCR | 0.412044913 | 2.04E-44 |
| MLST8 | AC009171.2 | 0.425371008 | 1.61E-47 |
| IL24 | LINC01857 | 0.426038493 | 1.12E-47 |
| CXCR4 | LINC01857 | 0.528760627 | 6.69E-77 |
| CASP1 | LINC01857 | 0.442059209 | 1.32E-51 |
| PRKCQ | LINC01857 | 0.670134234 | 3.21E-138 |
| PRKCQ | AC002091.1 | 0.407374722 | 2.31E-43 |
| CLN3 | LINC01569 | 0.444964463 | 2.44E-52 |
| MLST8 | LINC01569 | 0.473938917 | 4.51E-60 |
| ATG16L2 | ZKSCAN2.DT | 0.48201958 | 2.30E-62 |
| VMP1 | AL157838.1 | 0.437243592 | 2.11E-50 |
| GRID1 | AC134312.5 | 0.405886847 | 4.97E-43 |
| ATG16L2 | U73166.1 | 0.437645704 | 1.68E-50 |
| VMP1 | AC008115.3 | 0.458871004 | 5.86E-56 |
| ITGB1 | AC009065.4 | -0.464149853 | 2.24E-57 |
| BAX | AC009065.4 | 0.479486095 | 1.22E-61 |
| ATG101 | AC009065.4 | 0.406718183 | 3.24E-43 |
| CLN3 | AC009065.4 | 0.553379971 | 1.61E-85 |
| MLST8 | AC009065.4 | 0.555000142 | 4.10E-86 |
| GRID1 | LINC01711 | 0.40919545 | 9.02E-44 |
| DLC1 | MIR100HG | 0.401927907 | 3.74E-42 |
| TP63 | MIR100HG | 0.435126543 | 7.03E-50 |
| GRID1 | MIR100HG | 0.432001973 | 4.09E-49 |
| FOXO1 | MIR100HG | 0.546010142 | 7.27E-83 |
| ATG16L1 | AC008771.1 | 0.422358263 | 8.34E-47 |
| CTSB | LINC01094 | 0.498154029 | 3.95E-67 |
| SPHK1 | AC009041.2 | 0.425270466 | 1.70E-47 |
| CFLAR | AC004908.1 | 0.464856072 | 1.44E-57 |
| FOXO1 | AC002546.1 | 0.407802082 | 1.86E-43 |
| VMP1 | AL139407.1 | 0.494661351 | 4.47E-66 |
| CFLAR | AL139407.1 | 0.432559919 | 2.99E-49 |
| BAX | AL121832.2 | 0.417595284 | 1.08E-45 |
| VMP1 | AP005131.3 | 0.494929678 | 3.71E-66 |
| FOS | AC025259.3 | 0.563553418 | 2.69E-89 |
| VMP1 | AC124319.1 | 0.496584886 | 1.18E-66 |
| ITGB1 | AC069281.2 | -0.431248613 | 6.23E-49 |
| ULK3 | AC069281.2 | 0.436304705 | 3.60E-50 |
| BAX | AC069281.2 | 0.548152288 | 1.25E-83 |
| ATG101 | AC069281.2 | 0.468815077 | 1.19E-58 |
| MLST8 | AC069281.2 | 0.40628813 | 4.05E-43 |
| CFLAR | AC099343.2 | 0.442258204 | 1.18E-51 |
| FADD | AP000439.2 | 0.403010951 | 2.16E-42 |
| VMP1 | AC078778.1 | 0.420949189 | 1.79E-46 |
| CFLAR | AF131215.5 | 0.416320777 | 2.14E-45 |
| GRID1 | AL139393.2 | 0.51777862 | 2.80E-73 |
| VMP1 | AC087276.1 | 0.486893706 | 8.90E-64 |
| CFLAR | AC087276.1 | 0.415078871 | 4.13E-45 |
| VMP1 | AC124312.5 | 0.439193788 | 6.91E-51 |
| ATG16L2 | AP002807.1 | 0.431885548 | 4.36E-49 |
| ITGB1 | SPINT1.AS1 | -0.460292389 | 2.45E-56 |
| CLN3 | AC020765.2 | 0.416224195 | 2.25E-45 |
| MLST8 | AC020765.2 | 0.422300109 | 8.61E-47 |
| TP63 | MIR205HG | 0.405728531 | 5.40E-43 |
| BAX | AC007292.1 | 0.414626452 | 5.25E-45 |
| CFLAR | AC108010.1 | 0.410921975 | 3.67E-44 |
| PRKCQ | AC145098.1 | 0.474328714 | 3.51E-60 |
| CFLAR | AC145098.1 | 0.510482759 | 6.03E-71 |
| ATG16L2 | AC092119.2 | 0.469133656 | 9.74E-59 |
| GRID1 | AL513283.1 | 0.416611109 | 1.83E-45 |
| ULK3 | AP006284.1 | 0.44345324 | 5.89E-52 |
| ATG16L2 | AP006284.1 | 0.47771036 | 3.91E-61 |
| VMP1 | AL049840.1 | 0.450619384 | 8.62E-54 |
| CFLAR | AL049840.1 | 0.458531994 | 7.22E-56 |
| HGS | AC022211.2 | 0.424971439 | 2.01E-47 |
| BAX | AL355353.1 | 0.410525935 | 4.51E-44 |
| APOL1 | AC004847.1 | 0.449272017 | 1.92E-53 |
| CXCR4 | AC004847.1 | 0.488804947 | 2.45E-64 |
| FAS | AC004847.1 | 0.421360777 | 1.43E-46 |
| CASP1 | AC004847.1 | 0.503642625 | 8.26E-69 |
| PRKCQ | AC004847.1 | 0.707758411 | 8.48E-161 |
| CFLAR | AC004847.1 | 0.440809836 | 2.73E-51 |
| CFLAR | AC009120.3 | 0.413391348 | 1.01E-44 |
| APOL1 | AC147651.3 | 0.472959401 | 8.47E-60 |
| CTSB | AC147651.3 | 0.405996822 | 4.70E-43 |
| RGS19 | AC147651.3 | 0.452334801 | 3.09E-54 |
| CASP1 | AC147651.3 | 0.514949529 | 2.28E-72 |
| PRKCQ | AC147651.3 | 0.525186894 | 1.04E-75 |
| CLN3 | TP53TG1 | 0.46562916 | 8.88E-58 |
| GRID1 | MEG3 | 0.436652282 | 2.95E-50 |
| FOXO1 | MEG3 | 0.435687274 | 5.11E-50 |
| ITGB1 | AP000695.1 | 0.439364386 | 6.27E-51 |
| GRID1 | AP000695.1 | 0.441996076 | 1.37E-51 |
| SPNS1 | AC108134.1 | 0.447461774 | 5.61E-53 |
| BAX | AC084125.4 | 0.402748327 | 2.47E-42 |
| MLST8 | AC084125.4 | 0.445148287 | 2.19E-52 |
| CDKN2A | AL513165.1 | 0.452569701 | 2.68E-54 |
| MYC | AL513165.1 | 0.421181936 | 1.58E-46 |
| ATG16L2 | CCDC18.AS1 | 0.456952162 | 1.89E-55 |
| DLC1 | MIR22HG | 0.435757837 | 4.91E-50 |
| FOXO1 | MIR22HG | 0.45184995 | 4.13E-54 |
| CCL2 | MIR22HG | 0.499640746 | 1.40E-67 |
| MLST8 | AC003965.1 | 0.428776731 | 2.47E-48 |
| ULK3 | AC016773.1 | 0.421059092 | 1.69E-46 |
| BAX | AC016773.1 | 0.502912037 | 1.39E-68 |
| ATG16L2 | AC016773.1 | 0.411300365 | 3.01E-44 |
| VMP1 | XIST | 0.453730305 | 1.33E-54 |
| CFLAR | AC062037.2 | 0.492525271 | 1.94E-65 |
| CDKN2A | AP000251.1 | 0.409639047 | 7.16E-44 |
| ITGB1 | AC006942.1 | -0.407761852 | 1.89E-43 |
| BAX | AC006942.1 | 0.400663872 | 7.09E-42 |
| CLN3 | AC006942.1 | 0.443337653 | 6.30E-52 |
| MLST8 | AC006942.1 | 0.461551744 | 1.13E-56 |
| CFLAR | AL157392.3 | 0.497332688 | 7.01E-67 |
| GAPDH | PAXIP1.AS2 | -0.440125516 | 4.04E-51 |
| ULK3 | AP001453.3 | 0.425268105 | 1.71E-47 |
| VMP1 | AC122129.1 | 0.406836015 | 3.05E-43 |
| ITGB1 | AP001189.3 | 0.43323939 | 2.04E-49 |
| DLC1 | AP001189.3 | 0.558978018 | 1.39E-87 |
| GRID1 | AP001189.3 | 0.604945318 | 4.18E-106 |
| FOXO1 | AP001189.3 | 0.516023801 | 1.03E-72 |
| VMP1 | DLEU2 | 0.405010902 | 7.79E-43 |
| CDKN2A | U62317.2 | 0.412313607 | 1.77E-44 |
| BAX | U62317.2 | 0.436364413 | 3.48E-50 |
| APOL1 | AL590764.1 | 0.487660743 | 5.31E-64 |
| CXCR4 | AL590764.1 | 0.462155777 | 7.74E-57 |
| RGS19 | AL590764.1 | 0.419062674 | 4.94E-46 |
| CASP1 | AL590764.1 | 0.562606392 | 6.11E-89 |
| PRKCQ | AL590764.1 | 0.652278699 | 1.25E-128 |
| IKBKE | AL590764.1 | 0.404977094 | 7.93E-43 |
| MLST8 | AL022341.1 | 0.419301389 | 4.34E-46 |
| ITGB4 | SOX9.AS1 | 0.426299092 | 9.69E-48 |
| CFLAR | AP003392.1 | 0.467591039 | 2.59E-58 |
| CFLAR | AC234775.3 | 0.413019516 | 1.22E-44 |
| VMP1 | AL391001.1 | 0.481228155 | 3.88E-62 |
| CTSD | AC068580.1 | 0.558718139 | 1.74E-87 |
| BAX | AC074212.1 | 0.504888101 | 3.40E-69 |
| GAPDH | AC092718.4 | 0.46458927 | 1.70E-57 |
| BIRC5 | AC092718.4 | 0.479560441 | 1.16E-61 |
| CDKN2A | AC092718.4 | 0.402451212 | 2.87E-42 |
| MLST8 | MAFG.DT | 0.427757498 | 4.34E-48 |
| VMP1 | AC114763.1 | 0.529668778 | 3.31E-77 |
| ATG16L2 | AP003419.3 | 0.491834006 | 3.12E-65 |
| CLN3 | AP003419.3 | 0.496330006 | 1.41E-66 |
| ATG16L2 | AC004923.4 | 0.41587217 | 2.71E-45 |
| CLN3 | AC106782.2 | 0.548200769 | 1.20E-83 |
| MLST8 | AC106782.2 | 0.53222 | 4.54E-78 |
| CFLAR | AL356356.1 | 0.42999155 | 1.26E-48 |
| VMP1 | AF178030.1 | 0.542550551 | 1.22E-81 |
| VMP1 | AC010201.2 | 0.419347956 | 4.24E-46 |
| ATG16L2 | AC011472.1 | 0.474171931 | 3.88E-60 |
| ATG101 | AC133552.5 | 0.417817866 | 9.62E-46 |
| CLN3 | AC133552.5 | 0.469255652 | 9.02E-59 |
| MLST8 | AC133552.5 | 0.528977403 | 5.66E-77 |
| VMP1 | AC109361.2 | 0.49396123 | 7.24E-66 |
| GAPDH | AL133355.1 | -0.401802742 | 3.99E-42 |
| VMP1 | AC022146.2 | 0.407596453 | 2.06E-43 |
| VMP1 | AP005131.7 | 0.525479381 | 8.35E-76 |
| BAX | YTHDF3.AS1 | 0.410939041 | 3.64E-44 |
| IL24 | AC004687.1 | 0.415072771 | 4.14E-45 |
| RGS19 | AC004687.1 | 0.424402246 | 2.74E-47 |
| PRKCQ | AC004687.1 | 0.534897178 | 5.54E-79 |
| PRKCQ | MIAT | 0.491007651 | 5.48E-65 |
| CFLAR | MIAT | 0.510304535 | 6.86E-71 |
| CFLAR | AC007566.1 | 0.441489067 | 1.84E-51 |
| CLN3 | AC020663.2 | 0.526944877 | 2.71E-76 |
| MLST8 | AC020663.2 | 0.59467236 | 1.05E-101 |
| ATG16L2 | LINC00893 | 0.50995975 | 8.82E-71 |
| BAX | FLJ42351 | 0.416006274 | 2.52E-45 |
| VMP1 | AC092794.1 | 0.482794499 | 1.38E-62 |
| VMP1 | MIR99AHG | 0.408196939 | 1.51E-43 |
| IL24 | AC004585.1 | 0.462099231 | 8.02E-57 |
| CXCR4 | AC004585.1 | 0.526897289 | 2.81E-76 |
| RGS19 | AC004585.1 | 0.400596144 | 7.33E-42 |
| CASP1 | AC004585.1 | 0.45413494 | 1.05E-54 |
| PRKCQ | AC004585.1 | 0.680742889 | 3.05E-144 |
| CFLAR | AC091185.1 | 0.474901057 | 2.42E-60 |
| VMP1 | AC138932.5 | 0.423506708 | 4.47E-47 |
| VMP1 | AC018809.1 | 0.42482051 | 2.18E-47 |
| CLN3 | AC108134.4 | 0.476837377 | 6.91E-61 |
| MLST8 | AC108134.4 | 0.481934665 | 2.43E-62 |
| CLN3 | AC106782.6 | 0.506654199 | 9.60E-70 |
| MLST8 | AC106782.6 | 0.42008897 | 2.84E-46 |
| CFLAR | AL731571.1 | 0.403237801 | 1.93E-42 |
| PTEN | AL078581.1 | 0.405640112 | 5.65E-43 |
| MLST8 | SNHG9 | 0.400415219 | 8.03E-42 |
| VMP1 | AC005899.6 | 0.494933742 | 3.70E-66 |
| VMP1 | AC245014.3 | 0.418197068 | 7.85E-46 |
| VMP1 | AC093484.4 | 0.414020533 | 7.22E-45 |
| CFLAR | AL512791.1 | 0.450458528 | 9.49E-54 |
| ITGB1 | AC104667.2 | -0.404108197 | 1.24E-42 |
| BIRC5 | AC016205.1 | 0.406263503 | 4.10E-43 |
| ATG16L2 | AC092171.4 | 0.447610025 | 5.14E-53 |
| ATG16L2 | RAD51.AS1 | 0.49589648 | 1.90E-66 |
| ULK3 | AC009065.8 | 0.40886623 | 1.07E-43 |
| ATG16L2 | AC009065.8 | 0.509067096 | 1.68E-70 |
| MLST8 | AC009065.8 | 0.415717767 | 2.94E-45 |
| VMP1 | AC025171.4 | 0.422949776 | 6.05E-47 |
| PRKCQ | AC022706.1 | 0.466067298 | 6.75E-58 |
| ITGB1 | AC112721.2 | 0.445628738 | 1.65E-52 |
| DLC1 | AL136084.3 | 0.451718977 | 4.47E-54 |
| GRID1 | AL136084.3 | 0.473834204 | 4.83E-60 |
| FOXO1 | AL136084.3 | 0.407976353 | 1.70E-43 |
| ITGB1 | AC010503.4 | -0.449681799 | 1.51E-53 |
| CLN3 | AC138696.2 | 0.438971987 | 7.85E-51 |
| SPNS1 | AL031600.1 | 0.521322508 | 1.96E-74 |
| ATG16L2 | AL031600.1 | 0.510756055 | 4.94E-71 |
| BAX | AL391244.1 | 0.456694678 | 2.22E-55 |
| ATG101 | AL391244.1 | 0.44843274 | 3.16E-53 |
| MLST8 | AL391244.1 | 0.463361821 | 3.66E-57 |
| GRID1 | AL049838.1 | 0.542936475 | 8.91E-82 |
| FOXO1 | AL049838.1 | 0.445261022 | 2.05E-52 |
| VMP1 | NPTN.IT1 | 0.45861235 | 6.87E-56 |
| CFLAR | NPTN.IT1 | 0.462898001 | 4.88E-57 |
| ATG16L2 | AC095057.3 | 0.411030386 | 3.47E-44 |
| PPP1R15A | AC020916.1 | 0.599951335 | 6.03E-104 |
| FOS | AC020916.1 | 0.53749161 | 7.08E-80 |
| CFLAR | AC253536.3 | 0.427962104 | 3.87E-48 |
| VMP1 | AP000692.1 | 0.414612918 | 5.28E-45 |
| CFLAR | AP000692.1 | 0.449099066 | 2.13E-53 |
| ITGB1 | AC008915.2 | -0.418670133 | 6.10E-46 |
| CHMP4B | AC008915.2 | 0.449308032 | 1.88E-53 |
| CLN3 | AC008915.2 | 0.544017063 | 3.70E-82 |
| MLST8 | AC008915.2 | 0.610323658 | 1.79E-108 |
| APOL1 | LINC02446 | 0.518192532 | 2.05E-73 |
| CASP1 | LINC02446 | 0.448842548 | 2.48E-53 |
| PRKCQ | LINC02446 | 0.535120826 | 4.64E-79 |
| GRID1 | AC093278.2 | 0.443236701 | 6.68E-52 |
| FOXO1 | AC093278.2 | 0.520313022 | 4.20E-74 |
| MAP1LC3C | AC093278.2 | 0.459790865 | 3.33E-56 |
| TP63 | ACTA2.AS1 | 0.517226983 | 4.22E-73 |
| FOXO1 | ACTA2.AS1 | 0.420771642 | 1.97E-46 |
| CDKN2A | MELTF.AS1 | 0.48467918 | 3.93E-63 |
| GRID1 | AC005291.1 | 0.534670667 | 6.62E-79 |
| DLC1 | MAGI2.AS3 | 0.523922797 | 2.74E-75 |
| GRID1 | MAGI2.AS3 | 0.506992784 | 7.53E-70 |
| FOXO1 | MAGI2.AS3 | 0.700453894 | 3.88E-156 |
| CFLAR | MAGI2.AS3 | 0.413942844 | 7.53E-45 |
| CTSD | AC068580.3 | 0.684616581 | 1.66E-146 |
| ITGB4 | LINC00511 | 0.485273438 | 2.64E-63 |
| CDKN2A | LINC00511 | 0.447794855 | 4.61E-53 |
| EGFR | AC108673.2 | 0.407744116 | 1.91E-43 |
| ULK3 | AC073335.2 | 0.427754603 | 4.34E-48 |
| ITGB1 | AC008610.1 | -0.428322673 | 3.17E-48 |
| BAX | AC008610.1 | 0.433731847 | 1.55E-49 |
| BAX | AC005034.5 | -0.400179211 | 9.05E-42 |
| VMP1 | LINC01087 | 0.492695883 | 1.73E-65 |
| CFLAR | AC108449.2 | 0.457254257 | 1.58E-55 |
| VMP1 | AC083798.2 | 0.438239983 | 1.19E-50 |
| VMP1 | AC024145.1 | 0.443086547 | 7.29E-52 |
| BAX | AC083880.1 | 0.421825476 | 1.11E-46 |
| VMP1 | AL049840.5 | 0.514074011 | 4.35E-72 |
| CFLAR | AL049840.5 | 0.433185037 | 2.10E-49 |
| BIRC5 | AC012073.1 | 0.449044647 | 2.20E-53 |
| CDKN2A | AC012073.1 | 0.509543256 | 1.19E-70 |
| VMP1 | AC037198.1 | 0.508449168 | 2.63E-70 |
| VMP1 | AP002907.1 | 0.426849645 | 7.16E-48 |
| IL24 | TRBV11.2 | 0.402040057 | 3.54E-42 |
| APOL1 | TRBV11.2 | 0.415527964 | 3.25E-45 |
| CXCR4 | TRBV11.2 | 0.474712269 | 2.74E-60 |
| CASP1 | TRBV11.2 | 0.490760939 | 6.49E-65 |
| PRKCQ | TRBV11.2 | 0.70683133 | 3.37E-160 |
| CDKN2A | AC006329.1 | 0.498135037 | 4.01E-67 |
| MYC | AC006329.1 | 0.561608363 | 1.45E-88 |
| ULK3 | AL109811.3 | 0.401351048 | 5.01E-42 |
| ATG16L2 | AL109811.3 | 0.489495981 | 1.53E-64 |
| APOL1 | USP30.AS1 | 0.652334019 | 1.17E-128 |
| FAS | USP30.AS1 | 0.417971312 | 8.86E-46 |
| RGS19 | USP30.AS1 | 0.420401192 | 2.40E-46 |
| CASP1 | USP30.AS1 | 0.625724831 | 1.63E-115 |
| PRKCQ | USP30.AS1 | 0.498047697 | 4.26E-67 |
| IKBKE | USP30.AS1 | 0.441558928 | 1.77E-51 |
| GRID1 | LINC02544 | 0.432159816 | 3.74E-49 |
| ATG16L2 | SH3BP5.AS1 | 0.476707178 | 7.52E-61 |
| BAX | SNHG25 | 0.400194499 | 8.98E-42 |
| VMP1 | AL355075.2 | 0.437814473 | 1.52E-50 |
| CFLAR | AL355075.2 | 0.405571817 | 5.85E-43 |
| ATG16L2 | AC132192.2 | 0.457434686 | 1.41E-55 |
| ITGB1 | MSC.AS1 | 0.455170849 | 5.59E-55 |
| FOXO1 | MSC.AS1 | 0.479794487 | 9.99E-62 |
| ATG16L2 | AC005785.1 | 0.453707927 | 1.35E-54 |
| SPNS1 | AC009133.1 | 0.455485335 | 4.62E-55 |
| CHMP4B | CH17.340M24.3 | 0.411288774 | 3.03E-44 |
| CLN3 | CH17.340M24.3 | 0.484125096 | 5.68E-63 |
| MLST8 | CH17.340M24.3 | 0.416341852 | 2.11E-45 |
| CFLAR | AC093110.1 | 0.443199231 | 6.82E-52 |
| ITGB1 | AC005291.2 | 0.448677957 | 2.73E-53 |
| DLC1 | AC005291.2 | 0.403707293 | 1.52E-42 |
| GRID1 | AC005291.2 | 0.585506812 | 6.55E-98 |
| FOXO1 | AC005291.2 | 0.446219988 | 1.17E-52 |
| ERBB2 | AC006449.5 | 0.408863 | 1.07E-43 |
| MYC | VPS9D1.AS1 | 0.451972034 | 3.84E-54 |
| BAX | AC092803.2 | 0.411605167 | 2.57E-44 |
| BAX | AL390719.2 | 0.421826627 | 1.11E-46 |
| VMP1 | AP005131.2 | 0.461624028 | 1.08E-56 |
