# Supplementary material for: A Signature of Autophagy-Related Long Non-coding RNA to Predict the Prognosis of Breast Cancer
Source: Front Genet. 2021 Mar 16;12:569318. doi: 10.3389/fgene.2021.569318 (PMC8007922; doi:10.3389/fgene.2021.569318)
Supplement: Supplementary file 2 [file Table_2.DOCX]

Table 2. 18-autophagy related lncRNAs associated with prognosis.

| Gene | Coef |
| --- | --- |
| LINC01614 | 0.010385 |
| LINC02169 | 0.007272 |
| AP003119.3 | 0.046419 |
| AC109826.1 | 0.015188 |
| LINC01235 | 0.009169 |
| AC098484.1 | 0.143748 |
| LIPE.AS1 | -0.20838 |
| AL451085.2 | -0.03909 |
| LINC00667 | -0.09278 |
| TFAP2A.AS1 | -0.11147 |
| AC002398.1 | -0.05476 |
| PDCD4.AS1 | -0.08065 |
| AC061992.1 | -0.12714 |
| AC110619.1 | -0.03032 |
| ST8SIA6.AS1 | 0.02426 |
| AL512625.2 | -0.08938 |
| MIR4435.2HG | 0.100851 |
| AC147067.2 | 0.129416 |
