# Supplementary material for: A Signature of Autophagy-Related Long Non-coding RNA to Predict the Prognosis of Breast Cancer
Source: Front Genet. 2021 Mar 16;12:569318. doi: 10.3389/fgene.2021.569318 (PMC8007922; doi:10.3389/fgene.2021.569318)
Supplement: Supplementary file 3 [file Image_1.pdf]

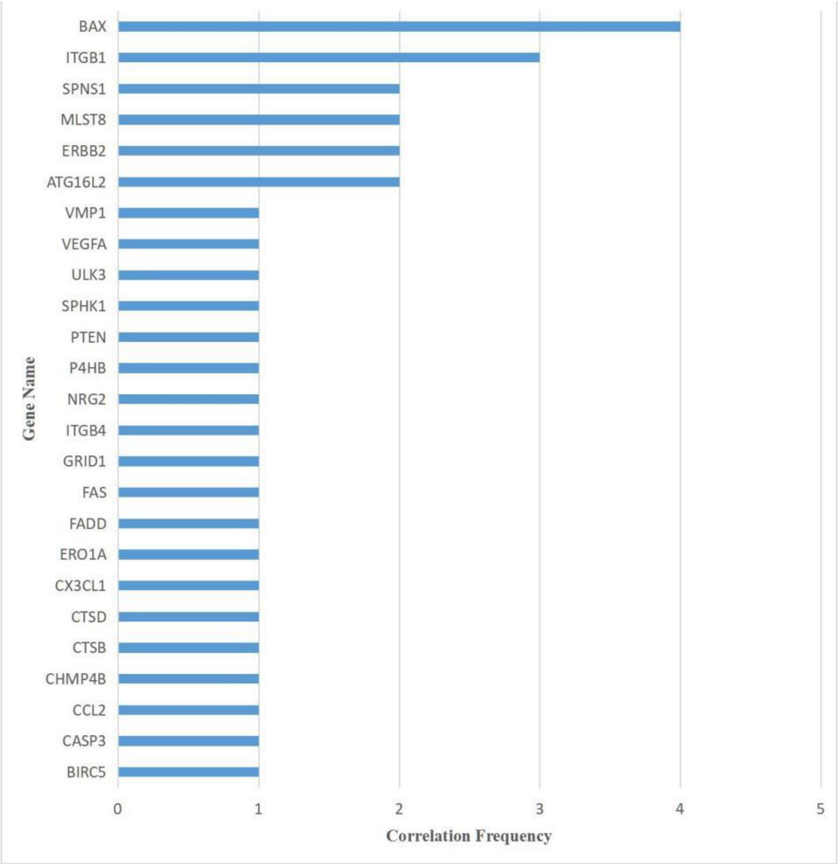

Fig S1. The correlation frequency between autophagy-related genes and 18 prognosis-associated lncRNAs.
